# An Overview of Tickborne Infections in Pregnancy and Outcomes in the Newborn: The Need for Prospective Studies

**DOI:** 10.3389/fmed.2020.00072

**Published:** 2020-03-06

**Authors:** John S. Lambert

**Affiliations:** ^1^Infectious Diseases Department, Mater Misericordiae University Hospital, Dublin, Ireland; ^2^Infectious Diseases Department, Rotunda Maternity Hospital, Dublin, Ireland; ^3^School of Medicine, University College Dublin, Dublin, Ireland

**Keywords:** neurodegenerative diseases, congenital Lyme disease, autism, tick borne infections in pregnancy, vertical transmission

## Abstract

Tick-borne infections are an ever-increasing issue internationally, many factors contribute to this including a changing climate. Pregnant women represent the single largest vulnerable group in populations due to a relative immune deficiency status. Infections in pregnant women have the added gravity of potential infection in the developing fetus which may have catastrophic consequences including death *in-utero* or lifelong debilitation. Currently there is a paucity of data surrounding tick-borne infections in pregnancy and long-term outcomes for mother and infant for conditions like Lyme disease and co-infections. At present there are no established international surveillance systems to identify and gain understanding of these infections in pregnancy. Furthermore, the removal of Congenital Lyme Disease from ICD-11 codes hampers dialogue and characterization of borreliosis in pregnancy and stifles future developments of this understudied domain. This review makes the case for further study and re-opening a dialogue of tick-borne infections in pregnancy.

## Introduction

Approximately 17% of all infectious diseases are vector borne with just over 50% of the world's population at risk at any time to one of these diseases (1). Those spread by vectors within the Insecta kingdom include mosquitoes, ticks, and flies. The focus of this review is on tick borne infections in pregnancy.

Pregnant women represent the single largest vulnerable population in society. Infections in this group not only impact the mother but have the added gravity of impacting the unborn fetus during the most fragile time of human development and can result in catastrophic lifelong changes in the unborn and also intra uterine death. This risk is compounded by immune modulation in pregnancy with, reduced CD/CD8 cells, decreasing cytotoxic T cells, and a shift from Th1 to Th2 Helper T Cells all increasing susceptibility to infection (2, 3). Overall there is poor understanding of infection and treatment of infection in pregnancy. The complex interactions at the materno-fetal interface are poorly understood. The role of the placenta as both a protective barrier from infection but also from maternal immune recognition while also supplying the fetus with all the essential nutrition for human development needs much more research. The spectrum of disease severity and presentation of tick-borne congenital infections from classic and well recognized syndromes to insidious atypical presentations that emerge after delivery in the developing child will be hypothesized and discussed based on recent published data.

Perinatal outcomes following infections acquired during pregnancy can range from minor self-limiting illnesses, to pregnancy loss by spontaneous abortion, invasive fetal infection, and can sometimes result in congenital syndromes. The timing of fetal infection *in utero* may determine the extent of disease manifestations and the outcome to the unborn child. In essence infections during the first trimester during development and folding of the neural tube and early brain development can result in catastrophic developmental defects. Certain infections may predispose the pregnancy to preterm labor and pre-term delivery, with adverse outcomes secondary to prematurity. Other infecting organism can have a direct effect, there is also the aberrant host immune response to these invading pathogens, and subsequent immunological damage. Thus infections in pregnancy can have a wide variety of outcomes, depending on the timing of infection, the type of infection, the interaction between the infecting organism and the immune system, and indeed certain host factors.

Infections can additionally be transmitted in the peri-partum period from mucosal exposure, and post-partum through breast feeding; such infections may manifest themselves immediately or in the later post-partum period, or even later in childhood and indeed extending into adolescence and adulthood. This time delay makes it much more difficult to link the original congenital infection with delayed complications and ultimately adverse medical outcomes for the offspring.

## Tick Borne Infections in Pregnancy

Hard-shell tick-borne infections primarily affect northern hemisphere temperate climates but have been found on all continents including Australia. Epidemiologically with rising global temperatures diseases like *Lyme borreliosis* are rising in incidence in Europe and north America, as ticks have a longer feeding season (4). Beyond the issue of “global warming” humans live “closer” to animals, and changes in our planet have resulted in an increase in zoonoses worldwide. As more humans are being bitten and infected, a significant percentage of these humans are women of child-bearing age, and indeed some are already pregnant.

### Lyme Disease

Lyme disease (LD) was first officially described in the State of Connecticut (Old Lyme), when a case series of children with juvenile arthritis were found to have spirochetal illness in 1977 (5). *Borrelia burgdorferi* was identified as the causative organism whose name is also now also used to describe a larger *Lyme borreliosis* complex (*Borrelia burgdorferi sensu lato*) which include *Borrelia burgdorferi sensu stricto, borrelia garinii, borrelia afzelii, borrelia miyamoti* amongst others. Vectors of Lyme are hard shelled ticks *ixodes scapularis* and *ixodes pacificus* in North America, *ixodes ricinus* in Europe and *ixodes persulcatus* in Asia. Ticks are also vectors for other disease like Ehrlichia, Rickettsiae, Bartonella, and Babesia, and often more than one infection can be spread by a tick at the same bite (called co-infections). Nymphal ticks that feed on small mammals and birds are the most transmissible of Lyme to humans. Infected ticks in endemic areas can have a wide range of prevalence's, ranging from 6-15% in Ireland, to over 50% in many EU countries and in the USA.

Vertical transmission of LD was first suspected in 1983 in a case that described arthritis in amother. Spirochetes were visualized on a blood film of the newborn who had presented with hyperbilirubinemia. However, no Lyme or syphilis serology was performed in this case (6), limiting conclusions. The first confirmed case with positive Lyme serology was described in 1985 in a 28-year-old mother who had acquired Lyme in the first trimester, who had a erythema chronicum migrans (ECM) rash, and delivered at 35 weeks. Symptoms consistent with LD developed in the mother post-delivery and her LD IFA was positive 1:128. The child died of congenital heart disease and autopsy showed spirochetes infiltrating the spleen, kidneys, and bone marrow, but were not found in cardiac tissue (7). A report by MacDonald successfully demonstrated *Borrelia burgdorferi* in the myocardium using an immunohistochemical technique (8).

In the following years a number of case reports present compelling immunohistological evidence of spirochetaemia in stillbirths where mothers had clinical and/or laboratory confirmed LD; confirming the vertical transmission of *B. burgdorferi* (8, 9). Evidence of clinical LD has been seen in infants in some instances: a 3 week old who developed a skin rash post-partum was found to have *B burgdorferi* isolated from biopsy of these skin specimens (10).

A case of neonatal LD was reported whereby Borrelia specific antibodies were discovered in the spinal fluid of an infant with documented neurologic dysfunction. The mother who had LD infection in her second trimester had been treated with oral antibiotics and was reported as being seronegative at the time of delivery (11). A case from Germany described an infant with neonatal onset of maculopapular skin rash, hepatosplenomegaly, anemia, and fever, followed by progressive multi-system manifestations including protruding eyes, bilateral knee arthritis, axillary and inguinal lymph nodes, growth impairment, and developmental delay. Elevated antibody titres against Borrelia were found in the child's serum; her mother, who had no clinical manifestations, also had positive ELISA titres (12). A case review of 19 women with LD in pregnancy reported adverse events in 5 cases of fetuses, suggesting the possibility of congenital LD (13).

Other suggestions of transplacental transmission pregnancy comes from studies of placental tissue tested post-delivery in mothers with LD; in one study of 60 mothers found to have antibodies against Borrelia, 5% had evidence of spirochetes in placenta tissue using silver stain. Two of 3 were PCR positive for *B burgdorferi* (14).

A study performed by Strobino et al. of over 2,000 women from an endemic region who 1had positive LD serology were compared to a Lyme negative cohort. Worse outcomes when comparing fetal deaths, pre term delivery and congenital abnormalities were not seen. Furthermore no risk of adverse outcomes was reported in women with reported tick exposure. In this study only 11 women had positive LD serology, 5 of whom had previously documented LD and who had received treatment (15). It is important to note that congenital defects in babies at 6-month follow-up was the only study marker of adverse outcome in newborns. There was no direct detection testing of placentas or of cord blood of babies born to these seropositive women Longitudinal health monitoring, serial serologies in newborns was not performed. The authors reported “a statistically significant association between past miscarriages and history of tick-bite” and “a significant association between having had a tick bite within 3 years of conception and congenital defects.” Authors also noted that the incidence of cardiac defects was twice as high in children born to mothers residing in towns with a high LD endemicity rate vs. low endemic areas. The authors also acknowledged that their study was underpowered and “the number of women was too small to draw conclusions about the risk of having a child with a congenital malformation if a woman is seropositive.”

A recent review on congenital tick-borne diseases by Jasik et al. opines, “it is possible that B. burgdorferi has a high ability to penetrate mammalian placentae due to its ability of active movement, antigenic and morphological variation, and many other features, which causes diagnostic difficulties and problems. In cases of intrauterine fetal infections among patients with Lyme disease, symptoms are not homogeneous. This suggests that *B. burgdorferi* s.l. is transmitted trans-placentally and may play an important role in the spreading of these pathogens.” Authors also acknowledge “the ability of long-term survival of *B. burgdorferi* s.l. in tissues and spreading of spirochetes in the body despite antibiotic treatment can contribute to intergenerational spread of Lyme disease” (16).

Most recently in 2018, Waddell et al. performed a systematic review of gestational LD and identified 59 cases between 1969 and 2017. Twelve cases report miscarriage or fetal death, 8 report new born death and 16 report other abnormalities post-delivery including syndactyly, respiratory distress, hyperbilirubinemia. One case described complete features of clinical and laboratory results consistent with vertical transmission of LD (17). They also summarized epidemiological studies comparing pregnant women in endemic areas with features or serology to non-Lyme pregnancies; their conclusion was that rates of adverse outcomes were not increased. There are discrepancies in the findings and interpretation of studies from the Waddell “systematic review” compared to other publications and reviews on this subject; questioning the accuracy of the term “systematic” in the title of their publication.

The literature on “Congenital Lyme” is at present incomplete due to lack of intensive investigations, and lack of longitudinal follow up of exposed infants, as has been done for another spirochete, syphilis. There is no doubt that congenital infection occurs with Borrelia; whether a congenital syndrome occurs as a result of this *in utero* infection remains to be further investigated.

Treatment of LD in pregnancy is complicated as doxycycline, the mainstay of treatment in non-pregnant adults, holds FDA class D in pregnancy due to disruption of teeth and bone during development. ca. Second line treatment with amoxicillin is advised in pregnancy, and recommendations suggest same treatment duration (18). Treatment of gestational LD has been associated with reduced adverse outcomes for the fetus (11%) vs. women not treated for infection in pregnancy (50%), which indicates some adverse outcomes for untreated gestational LD (17). A 2010 study authored by Lakos et al. reported adverse outcomes in parentally treated (12%), orally treated (31.6%) and of untreated women (60%) with LD during pregnancy (19). Some clinicians report preferential use of IV ceftriaxone 2G daily for 14 days for pregnant women with ECM, reporting a positive outcome in pregnant women and also good pregnancy outcomes (19–21).

### Ehrlichiosis

Ehrlichiosis is characterized by two separate genetically linked organisms with similar clinical presentations; *Anaplasma phagocytophilum* and *Ehrlichia chaffensis*. Both are gram negative obligate intracellular organisms. *A phagocytophilum* causes Human Granulocytic Anaplasmosis (HGA) and *E. chaffensis* causes human monocytic ehrlichiosis (HME). Both organisms are spread by the hard shelled ixodes ticks similar to LD, *I. scapularis* in eastern and upper mid-western United States and *I. pacificus* in western United States.

#### HGA

HGA is clinically characterized by flu like illness, leukopenia, thrombocytopenia, transaminitis, raised alkaline phosphatase, and raised LDH, with symptoms following outdoor activity. Morulae and intracellular inclusion are characteristically seen within neutrophils on Wright or Giemsa stain. IFA with 4-fold increase in antibodies is the diagnostic test of choice but peripheral smear and serum PCR can be more sensitive in early disease, performed before initiation of antibiotics. Although poorly described in pregnancy some case reports have shown infections can be treated successfully (22). Cases of miscarriage have been reported in patients treated for HGA with doxycycline (23). Vertical transmission have been reported in one mother who had tick exposure one week prior to delivering (24), another case series also reports vertical transmission in 1 of 6 women, no cases were seen in individuals treated with either rifampicin or doxycycline. HGA appears to have a mild course in pregnancy with no major adverse outcomes seen (25).

#### HME

HME although closely linked to HGA has some distinct features that differentiate it from HGA. The vectors for this bacterium are the lone star tick and the amblyomma tick. IFA is unreliable as a diagnostic tool in this instance. Morulae are seen in monocytes but with low frequency 1–20%. Only one adverse outcome has been reported in pregnancy, where a mother developed appendicitis and was treated with doxycycline. Both mother and baby had good long term outcomes (26).

### Babesiosis

Babesiosis, primarily caused by *Babesia microti* in humans is an intra-erythrocytic protozoal infection spread by Ixodes hard shelled ticks. When acquired patients are commonly co-infected with *Lyme borreliosis* and Anaplasma. In Europe *B. divergens* is the most common species, infection in humans is less commonly described compared to the US. Babesia is the most common transfusion related infection reported to the FDA (27). Clinical characteristics include fever, malaise, chills, jaundice, conjunctival hemorrhage, organomegaly, mild to moderate haemolytic anemia, thrombocytopenia. Diagnostics include PCR and wright giemsa stain which displays a characteristic “maltese cross” appearance of tetrads of merozoites within red cells. IFA, serology and ELISA are also used in diagnosis. Severity of disease is dependent on level of immune competence and the disease can progress to heart failure, ARDS, liver failure, renal failure, splenic rupture, and can carry mortality up to 20%.

As pregnancy is a relative immunocompromising state severe Babesia infections in pregnancy have been seen. Furthermore cases of vertical transmission, although rare, have been described. A congenital syndrome of fever, thrombocytopenia and anemia requiring transfusion is plausible. In one review of 9 cases, 2 were occult infections in mothers also infected with LD (28). Babesia can mimic HELLP (hemolytic anemia, elevated liver enzymes, and low platelets) syndrome in pregnancy. Patients from endemic areas, or who may have had blood transfusions from endemic areas should be investigated for Babesiosis. The first line treatment is atovaquone and azithromycin for mild to moderate disease and intravenous clindamycin and quinine for severe disease (18). Cases of use of clindamycin and primaquinee as first line therapy for mild and moderate disease have been reported as they have better placental penetration and potentially could reduce transmission.

### Tick Borne Encephalitis (TBE)

TBE is a neurotropic flavivirus spread by the same ticks as LD in Continental Europe and Asia. *I. ricinius* and *I. persulcatus* have the same small mammal reservoirs; TBE can also be spread by contaminated raw milk, particularly from goats (29, 30). The initial phase is characterized by non-specific viral prodrome, followed by a period without symptoms. A second phase after 4–5 weeks is characterized by neurological sequelae; meningitis, meningoencephalitis, radiculitis, myelitis, and paralysis. A case report of infection in the third trimester of pregnancy resulted in self-limiting illness with an uncomplicated spontaneous vaginal delivery. The TBE antibody was negative in the healthy neonate (31). No cases with evidence of vertical transmission have been seen. An inactivated vaccine is available; vaccine should be administered before pregnancy in those at risk; administration in pregnancy should only be considered when deemed necessary and an appropriate risk/benefit ratio is made (32).

### Relapsing Fever

While tick borne Borreliosis tends to focus on Lyme disease specifically, relapsing fever borreliosis (RF) is a significant cause of morbidity worldwide (33). Within tick borne relapsing fevers, the main vectors are the “soft ticks” of the genus Ornithodoros; but some species are transmitted by the ixodid vectors or “hard ticks.” Few studies have been done on this group of bacteria to further elucidate the interactions between host, tick, and pathogens. In pregnancy, it is claimed that relapsing fever Borreliosis may cause up to 10–15% of neonatal deaths worldwide (34).

RF borreliosis in pregnancy has a spectrum of complications; decreases in birth weight and preterm delivery in mild cases, or severe damage with miscarriage or neonatal death in severe cases (35, 36). One case report describes pregnant women with mild RF symptoms, but ultimately fatal outcome to the baby, who succumbed within 30 h of delivery (37). Recent mouse studies have shown RF infection of the fetus, can cause intrauterine growth retardation as well as placental damage and inflammation. Impaired fetal circulation causes spirochete and erythrocyte interactions as well as lowered maternal hemoglobin, in addition to direct invasion of the placenta (38). Further prospective studies in humans are needed to confirm the animal studies done to date.

### Rickettsial Disease

Rickettsia are a genetically related group of intracellular cocco-bacillary proteobacteria that have a pan-global distribution and cause febrile illnesses of variable severity. They are spread through a number of vectors including ticks, lice, fleas, and mites. There are over 20 species that can be broadly separated into four groups; ancestral, spotted fever, typhus, and transitional (39). Fever and rash are common features which can make rickettsial diseases difficult to distinguish from other infections.

Publications of clinical outcomes in pregnancy are limited in general as with other vector borne infections and appear to be worse than in the general population.

#### Rickettsia Rickettseii

*Rickettsia rickettseii* is the most pathogenic species and causes Rocky Mountain Spotted Fever (RMSF), a febrile illness with mortality rates as high as 20–30% without treatment. In the United States the American dog tick, a hard-shelled tick, *Dermacentor variabillis* in central and eastern states and *Dermacentor andersoni* in Western United States are the most common vectors. This infection is also endemic to other western hemisphere countries; Canada, Colombia, Brazil, Argentina, Costa Rica, Panama, and Mexico (40). A classical triad of fever, headache and rash is only present in around 60–70% of patients by week 2 post inoculation. Malaise, nausea, vomiting, abdominal pain are also features of early infection with mean onset of symptoms of 7 days. It can be missed in patients especially in pregnancy as other more common infections are suspected. The rash classically starts as a blanching macular rash at wrists and ankles and progresses to a non-blanching petechial rash that can become more confluent and progress to purpura. Occasionally progression to peripheral gangrene necessitates amputation. Disease progression can be severe within days of onset and can result in multi organ dysfunction, hepatomegaly, confusion, meningismus, pneumonia, and disseminated intravascular coagulation.

Studies in pregnant women are limited but the disease does not appear to be more severe in pregnant women. Vertical transmission has not been described. A case series of 4 patients in Mexico treated with doxycycline had negative outcomes. All of the mothers survived and one child born by SVD at 36 weeks had an uncomplicated course. Three women in the first trimester had spontaneous abortions (41). The authors identify 10 patients in the literature including their four patients. Doxycycline was used in 5 cases, chloramphenicol in 3 and amoxicillin in 2. Maternal fatality occurred in 3 cases, one was complicated by amputation of digits due to gangrene and the remaining cases were uncomplicated. Three neonates died post-partum, three miscarriages, one neonate had transient hyperbilirubinemia, and three uncomplicated outcomes. In the two cases where amoxicillin was administered resulted in fatality for both mother and fetus.

## How to Improve Our Understanding of the Impact of Tick-Borne Infections in Pregnant Women and Their Infants

From our review of the current literature on tickborne infections in pregnancy, we have identified a paucity of well-designed prospective studies and little investment in the accurate surveillance and monitoring of these infections worldwide. An independent group called the “ad hoc Committee for Health Equality in ICD11 Borreliosis Codes” was established in December 2015 to update the ICD11 codes for borreliosis diseases. One of the requests from this ad hoc Group was to have Congenital Lyme Disease instated as a “stand-alone” code and indeed in June of 2018 the WHO provided a provision code 1C1G.2 for Congenital Borreliosis. Having such a code would assist in the ability of researchers and advocates to petition for better studies and better funding to develop prospective studies and monitoring of women in pregnancy at risk and infected with tickborne infections; and longitudinally following up affected and infected children born to these mothers.

1C1G.2 Congenital *Lyme borreliosis* was removed in a very non-transparent manner from the ICD11 on December 17, 2018. Correspondence from a member of the ICD11 Medical and Scientific Advisory Committee of the WHO (MSAC) to an ad hoc member stated, “*This was in response to a request for the removal of Congenital Lyme borreliosis by the Public Health Agency of Canada (PHAC)…”*. Further communication from the WHO stated that there was no need for a “stand-alone” ICD code for Congenital Lyme as there was no recognized “congenital syndrome.” However, such an argument is lacking in scientific credence, and congenital malaria has a stand-alone ICD code, despite not having a recognized “congenital syndrome.” Despite multiple petitions and communications from members of the ad hoc Group and European MEPs and MPs to get clarification on the reason for “non transparent” deletion of the code from the proposed ICD 11, no response has been received from the responsible members of the WHO.

## What are the Consequences of “Missed” Lyme Diagnoses to the Unborn Child?

One unknown but plausible explanation for Autism Spectrum Disorders (ASD) is the possibility of a vertically transmitted infection in pregnancy. Bransfield et al. raise this issue and identify 24 infections and co-infections which may be contributing factors in the development of ASD in early childhood (42). Should this be the case he poses many unanswered questions; is the main mode of acquisition primary infection in infancy or from vertical transmission? Is the etiology of the disease caused by direct infection of nervous tissue or from a secondary immune response to infection, or both?

The evidence for a link between Lyme/tick-borne diseases (LY/TBD) and neuropsychiatric diseases in childhood has been raised from studies from the USA. Of the twenty states that reported the highest occurrence of Autistic Disorder per 10,000 people; fifteen reported a higher than average number of Lyme disease cases. Conversely, of the twenty states that reported the lowest incidence of Autistic Disorder per 10,000 people; zero reported a higher than average number of Lyme disease cases (43).

Although clinicians have previously suggested an association between Lyme disease and ASD, the first study provided a comprehensive case history review on the charts of 102 gestational LYD/TBD cases, and revealed that 9% had been diagnosed with autism and most were diagnosed with a broad spectrum of developmental disabilities. As a control, 66 mothers with Lyme disease who were treated with antibiotics prior to conception and during pregnancy; all gave birth to normal healthy infants (44) When children suspected with ASD were tested for Lyme, most studies demonstrate about 25% of ASD are infected with *Borrelia burgdorferi* (45).

It has been observed by Jones et al. that treatment of LYD/TBD during pregnancy can prevent the development of autism and other developmental disabilities associated with LYD/TBD (44). Another study has objectively demonstrated that antibiotic treatment can reduce ASD symptoms associated with LYD/TBD (43).

Another “mystery” childhood illness that has been attributed to congenital Lyme infection is Spinal Muscular Atrophy. Recent studies have suggested a connection between ALS and SMA (46).

## Summary

Tick borne infections are impacting on maternal and child health worldwide. The extent of this problem appears to be greater than current “status quo” acknowledges. Accurate data on the extent of tickborne infections worldwide is limited, and networks to monitor mothers and children following suspected tickborne exposure in pregnancy is essentially non-existent. Peer reviewed published articles on this disease area consists largely of case reports and small studies without adequate control. Many studies are retrospective in nature, which limits conclusions. However, these reports, limited in “quality” as they are, should represent a “red flag” to clinicians and public health officials within the health care system. And should be embraced, not ignored or discounted. The failure of recognition of Congenital Lyme both by clinicians caring for their patients, and by the WHO, who have failed to engage with the ICD 11 codes for Congenital Lyme, is a lost opportunity for better science and improved understanding. Such investment could result in improved maternal and child health, a clear purported declaration of the WHO, “no child left behind.” Science needs to prevail, and politics rather than science have to date won the day. And the children are losing.

## Author Contributions

JL: literature review, writing of the manuscript, and opinions expressed are those of the author.

### Conflict of Interest

The author declares that the research was conducted in the absence of any commercial or financial relationships that could be construed as a potential conflict of interest.
